# Air Pollution and Newly Diagnostic Autism Spectrum Disorders: A Population-Based Cohort Study in Taiwan

**DOI:** 10.1371/journal.pone.0075510

**Published:** 2013-09-25

**Authors:** Chau-Ren Jung, Yu-Ting Lin, Bing-Fang Hwang

**Affiliations:** Department of Occupational Safety and Health, College of Public Health, China Medical University, Taichung, Taiwan; Rikagaku Kenkyūsho Brain Science Institute, Japan

## Abstract

There is limited evidence that long-term exposure to ambient air pollution increases the risk of childhood autism spectrum disorder (ASD). The objective of the study was to investigate the associations between long-term exposure to air pollution and newly diagnostic ASD in Taiwan. We conducted a population-based cohort of 49,073 children age less than 3 years in 2000 that were retrieved from Taiwan National Insurance Research Database and followed up from 2000 through 2010. Inverse distance weighting method was used to form exposure parameter for ozone (O_3_), carbon monoxide (CO), nitrogen dioxide (NO_2_), sulfur dioxide (SO_2_), and particles with aerodynamic diameter less than 10 µm (PM_10_). Time-dependent Cox proportional hazards (PH) model was performed to evaluate the relationship between yearly average exposure air pollutants of preceding years and newly diagnostic ASD. The risk of newly diagnostic ASD increased according to increasing O_3_, CO, NO_2_, and SO_2_ levels. The effect estimate indicating an approximately 59% risk increase per 10 ppb increase in O_3_ level (95% CI 1.42–1.79), 37% risk increase per 10 ppb in CO (95% CI 1.31–1.44), 340% risk increase per 10 ppb increase in NO_2_ level (95% CI 3.31–5.85), and 17% risk increase per 1 ppb in SO_2_ level (95% CI 1.09–1.27) was stable with different combinations of air pollutants in the multi-pollutant models. Our results provide evident that children exposure to O_3_, CO, NO_2_, and SO_2_ in the preceding 1 year to 4 years may increase the risk of ASD diagnosis.

## Introduction

There is growing concern about the potential effect of air pollution on autism spectrum disorders (ASD). A group of neurodevelopmental disorders contains autistic disorder, Asperger's disorder, and pervasive developmental disorder not otherwise specified (PDD-NOS) is called ASD [1]. Abnormal development or impairment in social interaction, impaired verbal and non-verbal communication, and markedly restricted activity and interests are major characteristics of ASD [1], [2]. There is a large worldwide variation in the prevalence of ASD and there is also evidence that the prevalence has been increasing [3]. Both genetic and environmental factors play important roles in the aetiology of childhood ASD, and there is probably also genetic susceptibility to the effects of air pollution. Short-term changes in the occurrence of ASD are more likely influenced by changes in the environment than by change in the genetic pool. From preventive perspective, information on environmental, dietary, and behavioural factors is crucial. Identification of indicators of genetic susceptibility to environmental exposures could be useful from preventive point of view.

There is limited evidence that long-term exposure to ambient air pollution increases the risk of childhood ASD, but the role of different sources and components needs further elaboration [4]–[7]. Windham and colleagues in California studied hazardous air pollutants and ASD indicated an increased risk of autism with higher ambient air concentration of particularly metal (cadmium, mercury, and nickel), diesel particulate matter, and chlorinated solvents [4]. Kalkbrenner and colleagues examined children exposed to 35 hazardous air pollutants in North Carolina and West Virginia, and reported increased risk of ASD with increased concentration of methylene chloride, quinoline, and styrene [5]. Volk and colleagues found an association between early life living near a freeway within 309 m and risk of autism in California [6]. In addition, they reported traffic-related air pollution, nitrogen dioxide, particles with aerodynamic diameter less than 2.5 µm (PM_2.5_) and 10 µm (PM_10_) during pregnancy and during the first year of life were positively associated with autism [7]. Finding from previous studies of the effects of exposure to air pollution on the risk of ASD mainly has been cross-sectional or prevalent case-controls studies, where temporality constitutes problems.

We conducted a prospective 11-year population-based cohort study to access the association between postnatal exposure to air pollutants and newly diagnostic ASD in Taiwan. The design enabled us at the same time to verify an appropriate temporality between the hypothesized exposure and outcome and to eliminate the possibility that the presence of outcome would influence the assessment of exposure.

## Methods

### Study Design

We conducted a population-based cohort study by retrieving all individuals from the longitudinal health insurance database 2000 (LHID2000). The LHID2000 includes registration files and claim data of ambulatory care expenditures or inpatient expenditures for 1,000,000 individuals systematically and randomly selected from the year 2000 registry of beneficiaries of National Insurance Research Database (NHIRD). NHIRD comprised of detail health care information from more than twenty three million enrollees, representing more than 99% entire population in Taiwan. National Health Research Institutes (NHRI) confirmed there was no significant difference in the gender distribution, age distribution, newborn number every year, and average insurance payroll amount between LHID2000 and the NHIRD [8]. The accuracy of diagnosis of major diseases, such as acute coronary syndrome and ischemic stroke had been validated [9], [10]. Because the data was analyzed anonymously, the Institute Review Board specifically waived the need for consent by each subject. This study has been approved by the institute review board of China Medical University Hospital (CMU-REC-101-012), and it complied with the principles outlined in the Helsinki Declaration.

### Study Population

We included one millions individuals at the baseline in 2000. We focus on children that age less than 3 years at the baseline. The baseline study population constituted of 49,833 individuals. We conducted a prospective cohort study directed at all the individuals of the cohort from January 1, 2000 through December 31, 2010. The follow-up time began from each individual's first date of enrollment until the first diagnosis of ASD. In the present analyses, we excluded children, who had experienced ASD at the baseline (N = 4), and those who had missing information on air pollution (N = 756). Thus the final study population constituted a total of 49,073 individuals. There were 535 individuals (1.09%) lost to follow-up during the study period. The response rate is up to 98.91%.

### Outcome of interest

The database provides personal diagnosis codes according to *International Classification of Diseases, Ninth Revision, Clinical Modification* (ICD-9-CM). The bureau of National Health Insurance verify the validity and quality of diagnosis by random sampling a constant ratio of claims from every hospital each year and through strict review by an independent group of doctors [11]. To ensure the diagnostic validity of ASD in present study, only children with at least two consensus diagnosis of autistic disorder (code 299.0), Asperger syndrome (ICD-9-CM code 299.8), or PDD-NOS (ICD-9-CM 299.9)[12] between January 1, 2000 and December 31, 2010 that were selected as our outcome of interest. The newly diagnostic ASD was defined as the diagnosis code first appeared in the claims.

### Exposure assessment

Carbon monoxide (CO), nitrogen dioxide (NO_2_), sulfur dioxide (SO_2_) and PM_10_ monthly average data and Ozone (O_3_) monthly average of daily maximum value were obtained from 70 Taiwan Environmental Protection Agency (EPA) monitoring station on Taiwan's main island. All of these air pollutants measured hourly—CO by nondispersive infrared absorption, NO_2_ by chemiluminescence, O_3_ by ultraviolet absorption, SO_2_ by ultraviolet fluorescence, and PM_10_ by beta-gauge—and continuously.

The locations of the monitoring stations and air pollution sources were identified and managed by geographic information system (GIS) (ArcGIS version 10; ESRI, Redlands, CA, USA). The monitoring data were integrated into yearly point data and interpolated to pollutant surfaces using inverse distance weighting method (IDW). For the IDW approach, we used suitable spatial resolution (100 m) [13] and inverse squared distance (1/squared distance) weighted average of the three nearest monitors within 25 kilometer to compute yearly mean concentration. Then, the yearly air pollution data were assigned to individuals by post-code levels. The postal code typically corresponded to one block face in urban areas (mean±SD: 17±8.56 square kilometer) but was larger in rural areas (mean±SD: 154±104.39 square kilometer) with low population density. We averaged exposure to CO, NO_2_, O_3_, PM_10_, and SO_2_ over several years before newly diagnostic ASD: preceding 1 year, 2 years, 3 years, and 4 years.

### Covariates

The baseline age in 2000 and gender (male or female) of children were available from NHIRD and municipal-level socioeconomic status (SES) of parents was determined based on distribution of annual average incomes data from Directorate-general of Budget, Account, Statistics which was classified to four level: >75th percentile, 75th to 50th percentile, 50th to 25th percentile, and <25th percentile.

Some important comorbidities related to ASD were also included [14], [15]. We included anxiety (ICD-9-CM code 300.0), bipolar disorder (296.4–296.8), depressive disorder (296.2, 296.3 and 311), intellectual disabilities (317–319), obsessive-compulsive disorder (300.3), and phobic disorder (300.2). Besides, children were classified into preterm (765.0 or 765.1) and full-term (children without a code of 765.x) [16].

### Statistical methods

Extended Cox proportional hazards (PH) model incorporated time-dependent and time-independent variables as predictors that were used to investigate the association between air pollutants and ASD. For the analysis of time to newly diagnostic ASD, each individual's survey time was censored at the year when the person was quite insurance, or at the end of the follow-up. Any variable for a given individual change over time is called a time dependent variables [17]. We applied an extended Cox PH model: 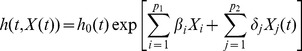
where a baseline hazard function *h_0_(t)* is multiplied by an exponential function which contain *X_i_*, time-independent variables, and *X_j_*, time-dependent variables. The *X(t)* is the entire collection of predictor [17]. Air pollutants such as CO, NO_2_, O_3_, PM_10_ and SO_2_ were treated as time-dependent variables in the Cox PH model, representing average exposure over several years before the newly diagnostic ASD: preceding 1 year, 2 years, 3 years, and 4 years. The children whose age of newly diagnostic ASD was younger than deductive years prior to newly diagnostic ASD were removed from analyses (e.g., we excluded the children whose age of newly diagnostic ASD was younger than four years old when assessing four years prior to first diagnosis of ASD).

We first applied chi-square tests and t-tests including the covariates listed above to identify covariates associated with newly diagnostic ASD and air pollution, which were subsequently included in Cox PH model investigating the association between air pollution exposures and ASD. Further, we also selected potential confounders *a priori* and included them into the final model.

We fitted one pollutant models and two pollutants models to estimate the effect average exposure of CO, NO_2_, O_3_, PM_10_ and SO_2_ on newly diagnostic ASD while controlling second pollutant in the model. The effect of each pollutant on the risk of newly diagnostic ASD was estimated as hazard ratio (HR) per 100-ppb change for CO, 10-ppb change for NO_2_ and O_3_, 10-µg/m^3^ change for PM_10_, and 1-ppb for SO_2_, along with their 95% confidence interval (CI). We also compared the risk of newly diagnostic ASD in four exposure categories based on the distribution of each pollutant representing high (>75th percentile), medium (75th to 50th percentile), low exposure (<50th to 25th percentile) and <25th percentile as the reference category. All analyses were performed with SAS version 9.2 for Windows (PROC PHREG).

## Results

Within the cohort of 49,073 children from January 1, 2000 to December 31, 2010, a total of 342 children developed ASD. In our study, most children developed ASD were male (83.2%), younger children (baseline age 1.01±0.95 years; age of newly diagnostic ASD 6.26±2.91 years) and with higher SES (≥1,095,192). The common comorbidities were intellectual disabilities (35.67% of children with newly diagnostic ASD), and anxiety (13.16%) (Table 1).

**Table 1:** Demographic data of the study cohort from January 1, 2000 to December 31, 2010.

Characteristics	ASD n = 342		non ASD n = 48,731		*P* a
	Total n	Column%	Total n	Column%	
Gender					<0.0001
Female	58	16.96	23,454	48.13	
Male	284	83.04	25,277	51.87	
Age (years) in 2000					0.0070
Mean±SD	1.01±0.95		1.15±1.01		
Age (years) of newly diagnostic ASD					<0.0001
Mean±SD	6.26±2.91		11.94±1.39		
SES					<0.0001
<913,847	52	15.20	11,59	23.79	
913,848-1,095,191	65	19.01	10,302	21.14	
1,095,192-1,209,362	114	33.33	16,113	33.07	
≥1,209,363	111	32.46	10,724	21.85	
Anxiety					<0.0001
Yes	45	13.16	750	1.54	
No	297	86.84	47,981	98.46	
Bipolar Disorder					0.1114
Yes	1	0.29	17	0.03	
No	341	99.71	48,714	99.97	
Depressive Disorder					0.0411
Yes	2	0.58	47	0.10	
No	340	99.42	48,684	99.90	
Intellectual Disabilities					<0.0001
Yes	122	35.67	641	1.34	
No	220	64.33	48,080	98.66	
Obsessive Compulsive Disorder					<0.0001
Yes	8	2.34	43	0.09	
No	334	97.66	48,688	99.91	
Phobic Disorder					0.0195
Yes	2	0.58	30	0.06	
No	340	99.42	48,701	99.94	
Preterm					<0.0001
Yes	22	6.43	1138	2.34	
No	320	93.57	47593	97.66	

Note: ASD, autism-spectrum disorders; SES, municipal-level socioeconomic status.

a χ^2^ tests for categorical variables; t-tests for continuous variables.

### Effect of air pollution before newly diagnostic ASD

We investigated the relationship between newly diagnostic ASD and average exposure over several years prior to newly diagnostic ASD. The adjusted HR of newly diagnostic ASD increased with raised concentration of O_3_ per 10-ppb change, the tendency was similar from preceding 1 year to 4 years, varying between 1.27 and 1.60 (Table 2). The risk of newly diagnostic ASD also positively associated with a 100-ppb change in CO (adjusted HR varying between 1.37 and 1.43) and 10-ppb change in NO_2_ (adjusted HR varying between 3.14 and 4.75). There is no association between the risk of newly diagnostic ASD and PM_10_ from preceding 1 year to 4 years (adjusted HR varying between 0.92 and 1.08). The risk of newly diagnostic ASD was positively associated with 1-ppb change of SO_2_ in the preceding 1 year and 2 years (adjusted HR varying between 1.13 and 1.18), but the effect estimates were decreased substantially in the preceding 3 years and 4 years (adjusted HR varying between 0.88 and 0.94) (Table 2). Because of consistent pattern of all pollutants, we therefore presented average exposure of CO, NO_2_, O_3_, PM_10_, and SO_2_ of preceding 1 year before newly diagnostic ASD in the subsequent analysis.

**Table 2:** Crude and adjusted hazard ratios (95% confidence interval) for newly diagnostic autism spectrum disorders (ASD) from single pollutant model over several years before newly diagnostic ASD.

	Crude HRs (95% CI)	Adjusted HRs (95% CI)
**O_3_ (10 ppb)** a		
Preceding 1 year	1.30 (1.19–1.42)	1.59 (1.42–1.78)
Preceding 2 years	1.34 (1.22–1.48)	1.60 (1.42–1.80)
Preceding 3 years	1.17 (1.07–1.29)	1.36 (1.21–1.52)
Preceding 4 years	1.12 (1.01–1.23)	1.27 (1.13–1.43)
**CO (100 ppb)** a		
Preceding 1 year	1.34 (1.29–1.40)	1.37 (1.31–1.44)
Preceding 2 years	1.37 (1.32–1.43)	1.40 (1.33–1.46)
Preceding 3 years	1.36 (1.30–1.42)	1.39 (1.32–1.46)
Preceding 4 years	1.36 (1.30–1.43)	1.43 (1.35–1.52)
**NO_2_ (10 ppb)** a		
Preceding 1 year	3.46 (2.80–4.28)	4.43 (3.33–5.90)
Preceding 2 years	3.49 (2.81–4.32)	4.75 (3.54–6.39)
Preceding 3 years	2.86 (2.29–3.58)	3.56 (2.60–4.87)
Preceding 4 years	2.70 (2.13–3.42)	3.14 (2.25–4.39)
**PM_10_ (10 µg/m^3^)** b		
Preceding 1 year	0.93 (0.86–1.02)	1.08 (0.97–1.19)
Preceding 2 years	0.90 (0.82–0.98)	1.02 (0.91–1.13)
Preceding 3 years	0.85 (0.77–0.94)	0.94 (0.84–1.05)
Preceding 4 years	0.83 (0.75–0.93)	0.92 (0.81–1.04)
**SO_2_ (1 ppb)** b		
Preceding 1 year	1.13 (1.06–1.20)	1.18 (1.09–1.28)
Preceding 2 years	1.09 (1.02–1.17)	1.13 (1.04–1.23)
Preceding 3 years	0.97 (0.89–1.05)	0.94 (0.86–1.04)
Preceding 4 years	0.94 (0.86–1.02)	0.88 (0.80–0.98)

Note: CO, carbon monoxide; NO_2_, nitrogen dioxides; O_3_, ozone; PM_10_, particles with aerodynamic diameter less than 10 µm; SO_2_, sulfur dioxide.

a Adjusted HRs were adjusted for age, anxiety, gender, intellectual disabilities, preterm, and SES.

b Adjusted HRs were adjusted for age, anxiety, gender, intellectual disabilities, obsessive compulsive disorder, phobia, preterm, and SES.

### Air pollution

**Figure 1:**
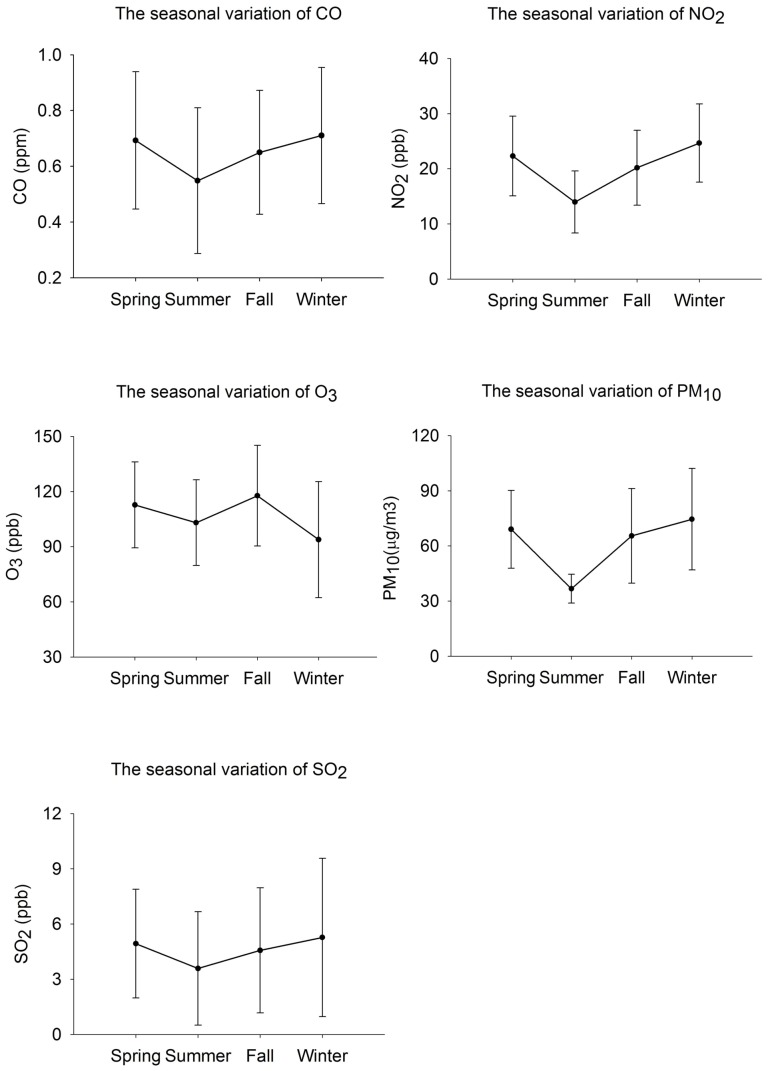
The seasonal variation of five main air pollutants during the study period.

The seasonal concentrations of O_3_ are higher in spring, summer, and fall, and lower in winter. The concentrations of CO, NO_2_, PM_10_, and SO_2_ are lowest in summer, and higher in both side (Figure 1). We examined the results of spearman correlation for average concentration of preceding 1 year before newly diagnostic ASD (Table 3). The concentration of CO was highly and positively correlated with NO_2_ (*r* = 0.95), which represent the common source of motor vehicles, and moderately correlated with SO_2_ (*r* = 0.32). O_3_ was highly correlated with PM_10_ (*r* = 0.65).

**Table 3:** Spearman correlation of average concentration of air pollutants of preceding year before end of follow-up.

	CO	NO_2_	O_3_	PM_10_	SO_2_
CO	1	0.95**	−0.05**	−0.13**	0.32**
NO_2_		1	−0.00	−0.02**	0.45**
O_3_			1	0.65**	0.22**
PM_10_				1	0.44**
SO_2_					1

Note: CO, carbon monoxide; NO_2_, nitrogen dioxides; O_3_, ozone; PM_10_, particles with aerodynamic diameter less than 10 µm; SO_2_, sulfur dioxide. ** P<0.0001.

### Air pollution and newly diagnostic ASD

In the single-pollutant model, the risk of newly diagnostic ASD was increased in relation to the O_3_ levels per 10-ppb changes (adjusted HR = 1.59; 95% CI 1.42–1.78). The adjusted HR was 1.72 (95% CI 1.20–2.47) for low, 2.55 (95% CI 1.72–3.78) for medium and 5.88 (95% CI 4.05–8.52) for high O_3_ exposure and shows an exposure-response pattern (Table 4). In the two-pollutant models, the effects estimates for O_3_ exposure were stable for the three different combinations of pollutants, varying between 1.53 and 1.57 (Table 5).

**Table 4:** Crude and adjusted hazard ratios (95% confidence interval) for newly diagnostic autism spectrum disorders (ASD) from single pollutant model.

	Crude HRs (95% CI)	Adjusted HRs (95% CI)
**O_3_** a		
10 ppb	1.30 (1.19–1.42)	1.59 (1.42–1.78)
<96.91 ppb	1 (Reference)	1 (Reference)
96.92–106.43 ppb	1.81 (1.27–2.58)	1.72 (1.20–2.47)
106.44–112.21 ppb	1.52 (1.05–2.18)	2.55 (1.72–3.78)
≥112.22 ppb	2.88 (2.07–4.00)	5.88 (4.05–8.52)
**CO** a		
100 ppb	1.34 (1.29–1.40)	1.37 (1.31–1.44)
<0.41 ppm	1 (Reference)	1 (Reference)
0.42–0.50 ppm	2.47 (1.47–4.15)	2.59 (1.53–4.37)
0.51–0.64 ppm	4.48 (2.76–7.29)	6.08 (3.65–10.13)
≥0.65 ppm	9.17 (5.78–14.54)	13.67 (8.14–22.97)
**NO_2_** a		
10 ppb	3.46 (2.80–4.28)	4.43 (3.33–5.90)
<15.80 ppb	1 (Reference)	1 (Reference)
15.81–18.92 ppb	1.27 (0.86–1.87)	1.26 (0.85–1.87)
18.93–22.76 ppb	1.52 (1.05–2.19)	2.02 (1.33–3.05)
≥22.77 ppb	3.89 (2.82–5.38)	4.87 (3.22–7.37)
**PM_10_** b		
10 µg/m^3^	0.93 (0.86–1.02)	1.08 (0.97–1.19)
<48.68 µg/m^3^	1 (Reference)	1 (Reference)
48.69–57.96 µg/m^3^	1.48 (1.11–1.96)	1.38 (1.04–1.84)
57.97–70.57 µg/m^3^	1.01 (0.74–1.36)	1.36 (0.98–1.91)
≥70.58 µg/m^3^	0.69 (0.49–0.97)	0.94 (0.64–1.37)
**SO_2_** b		
1 ppb	1.13 (1.06–1.20)	1.18 (1.09–1.28)
<3.34 ppb	1 (Reference)	1 (Reference)
3.35–3.90 ppb	0.68 (0.49–0.96)	0.72 (0.51–1.06)
3.91–4.66 ppb	0.89 (0.65–1.20)	0.95 (0.70–1.30)
≥4.67 ppb	1.53 (1.15–2.02)	2.03 (1.44–2.87)

Note: CO, carbon monoxide; NO_2_, nitrogen dioxides; O_3_, ozone; PM_10_, particles with aerodynamic diameter less than 10 µm; SO_2_, sulfur dioxide.

a Adjusted HRswere adjusted for age, anxiety, gender, intellectual disabilities, preterm, and SES.

b Adjusted HRs were adjusted for age, anxiety, gender, intellectual disabilities, obsessive compulsive disorder, phobia, preterm, and SES.

**Table 5:** Adjusted hazard ratios (95% confidence interval) of two pollutant mode for newly diagnostic autism spectrum disorders (ASD).

Air pollutant	CO+O_3_ a	CO+PM_10_ b	CO+SO_2_ b	NO_2_+O_3_ a	NO_2_+PM_10_ b	NO2+SO_2_ b	O_3_+SO_2_ b	PM_10_+SO_2_ b
O_3_ (10 ppb)	1.57 (1.40–1.77)			1.53 (1.35–1.73)			1.54 (1.37–1.73)	
<96.91 ppb	1 (Reference)			1 (Reference)			1 (Reference)	
96.92–106.43 ppb	1.15 (0.79–1.67)			1.22 (0.84–1.77)			1.79 (1.23–2.60)	
106.44–112.21 ppb	1.80 (1.20–2.70)			2.25 (1.50–3.38)			2.80 (1.89–4.13)	
≥112.22 ppb	4.05 (2.74–6.01)			4.93 (3.35–7.25)			4.95 (3.39–7.22)	
CO (100 ppb)	1.37 (1.30–1.43)	1.40 (1.33–1.46)	1.37 (1.31–1.44)					
<0.41 ppm	1 (Reference)	1 (Reference)	1 (Reference)					
0.42–0.50 ppm	1.83 (1.07–3.12)	3.16 (1.85–5.38)	2.63 (1.54–4.50)					
0.51–0.64 ppm	3.26 (1.91–5.56)	7.99 (4.69–13.62)	5.29 (3.13–8.95)					
≥0.65 ppm	8.66 (5.12–14.63)	18.87 (10.96–34.47)	13.46 (7.87–23.02)					
NO_2_ (10 ppb)				4.10 (3.04–5.55)	5.69 (4.18–7.23)	4.57 (3.39–6.16)		
<15.80 ppb				1 (Reference)	1 (Reference)	1 (Reference)		
15.81–18.92 ppb				0.75 (0.50–1.13)	1.51 (0.99–2.30)	1.33 (0.87–2.03)		
18.93–22.76 ppb				1.16 (0.75–1.79)	2.24 (1.43–3.49)	1.76 (1.13–2.72)		
≥22.77 ppb				2.81 (1.85–4.27)	6.33 (4.04–9.92)	4.73 (3.06–7.30)		
PM_10_ (10 µg/m^3^)		0.91 (0.81–1.02)			0.80 (0.70–0.91)			0.87 (0.75–1.00)
<48.68 µg/m^3^		1 (Reference)			1 (Reference)			1 (Reference)
48.69–57.96 µg/m^3^		0.95 (0.70–1.29)			0.90 (0.66–1.22)			1.01 (0.74–1.38)
57.97–70.57 µg/m^3^		0.74 (0.52–1.05)			0.98 (0.68–1.40)			1.21 (0.86–1.70)
≥70.58 µg/m^3^		0.44 (0.29–0.67)			0.54 (0.36–0.81)			0.43 (0.27–0.68)
SO_2_ (1 ppb)			1.10 (1.02–1.19)			1.00 (0.92–1.09)	1.08 (1.00–1.17)	1.28 (1.14–1.42)
<3.34 ppb			1 (Reference)			1 (Reference)	1 (Reference)	1 (Reference)
3.35–3.90 ppb			0.79 (0.56–1.12)			0.80 (0.55–1.15)	0.76 (0.54–1.07)	0.77 (0.54–1.09)
3.91–4.66 ppb			0.65 (0.47–0.90)			0.68 (0.49–0.95)	0.92 (0.67–1.25)	1.16 (0.83–1.63)
≥4.67 ppb			1.17 (0.81–1.68)			1.49 (1.02–2.18)	1.48 (1.04–2.12)	3.50 (2.28–5.36)

Note: CO, carbon monoxide; NO_2_, nitrogen dioxides; O_3_, ozone; PM_10_, particles with aerodynamic diameter less than 10 µm; SO_2_, sulfur dioxide.

a Adjusted HRs were adjusted for age, gender, SES, anxiety, and intellectual disabilities.

b Adjusted HRs were adjusted for age, anxiety, gender, intellectual disabilities, obsessive compulsive disorder, phobia, preterm, and SES.

The risk of newly diagnostic ASD was also associated with a 100-ppb change in CO (adjusted HR = 1.37, 95% CI1.31–1.44) (Table 4), and inclusion of both of the traffic-related pollutants and O_3_ did not change the effect estimate substantially (Table 5). The effect estimates for newly diagnostic ASD for CO were significantly elevated with an exposure-response pattern (adjusted HR low CO = 2.59, 95% CI 1.53–4.37; adjusted HR medium CO = 6.08, 95% CI 3.65–10.13; adjusted HR high CO = 13.67, 95% CI 8.14–22.97) (Table 4) and the estimates changed little when different second pollutants were added (Table 5). As for CO, the adjusted hazard ratio for 10-ppb change in NO_2_ for ASD in the single pollutant model were 4.43 (95% CI 3.33–5.90) (Table 4) and the estimates changed little when different second pollutants were added (Table 5). The risk of newly diagnostic ASD was elevated with an exposure-response pattern in the single pollutant model and inclusion both of the O_3_ and stationary fossil fuel combustion-related air pollutants (SO_2_ and PM_10_) did not change the effect estimate substantially (Table 5). The adjusted HR for 1-ppb change in SO_2_ for ASD in the single-pollutant model was 1.18 (95% CI 1.09–1.28), but did not show an exposure-response pattern (Table 4). Surprisingly, an inverse association between PM_10_ exposure and newly diagnostic ASD was found.

## Discussion

The risk of newly diagnostic ASD increased according to increasing O_3_, CO, NO_2_, and SO_2_ levels. The effect estimate indicating an approximately 59% risk increase per 10-ppb increase in O_3_ level, 37% risk increase per 10-ppb in CO, 343% risk increase per 10-ppb increase in NO_2_ level, and 18% risk increase per 1-ppb in SO_2_ level was stable with different combinations of air pollutants in the two-pollutant models. The results provide evidence that children exposure to O_3_, CO, NO_2_, and SO_2_ in the preceding 1 year to 4 years may increase the risk of newly diagnostic ASD. A negative or weak association between the risk of newly diagnostic ASD and PM_10_ was found.

There were four previous studies accessed the associations between air pollutants and ASD, but they provided inconsistent results. Exposure to Metal, diesel particulate matter, chlorinated solvents, quinoline, and styrene of ambient air during prenatal, perinatal, or early childhood may increase the risk of ASD [4], [5]. By examining the associations between distance to nearest freeway or major road and autism, Volk and colleagues found living near a freeway (less than 309 m) may increase the risk of autism [6], and furthermore, they reported that higher local estimates of traffic-related air pollution (traffic-related pollutant mixture include elemental carbon, CO, and NO_2_) and regional measures of PM_2.5_, PM_10_, and NO_2_ during pregnancy and first year of life would increase the risk of autism [7]. Consistently, the present study found results the risk of ASD increased with increasing concentration of traffic-related pollutants, such as CO and NO_2_. In addition, our results also found O_3_ and SO_2_ were significantly positive correlated with ASD. Oxidative stress may contribute to the pathogenesis of ASD through lipid peroxidation, reduced antioxidant activity, elevated nitric oxide level [18]–[23] and inflammation [24], [25]. Ambient air is a complex of variety individual pollutants that are free radical such as NO_2_or have the ability to drive free radical reaction such as O_3_ and particulates [26]. Carbon monoxide is 218 times of tendency to bind with Hemoglobin (Hb) than with oxygen [27]. When CO bind to Hb to form carboxyhemoglobin (COHb), then the oxygen carrying capacity of the blood decreases may cause tissue hypoxia. Hypoxia may deprive of usual supply of oxygen and damage cells of the heart, muscle, brain and nervous system [28], [29]. Children exposed to CO and then reoxygenation may generate oxidative stress and damage brain attribute to the increased production of partially reduced oxygen species (PROS) [30] or lipid peroxides arise from oxidative degradation of brain lipids [31]. Exposure to NO_2_ may induce the release of proinflammatory mediators from the lung [32]. Chronic lung inflammation may result in systemic inflammation that impacts blood vessel [33]. Cytokine derived from Systemic inflammation may also across the blood brain barrier [34] and active microglia [35]. Actively microglia may release secondary inflammation mediators and intensify neuroinflammation, then lead to brain damage [35]. Acute or chronic O_3_ exposure may generate oxidative stress that lead to brain lipid peroxidation [36], [37], neuronal morphological and ultrastructural changes [38], and memory deterioration [39]. Further, expose to low doses of O_3_ can develop progressive dopaminergic neuronal death in the substantia nigra of animal model [40]. Superoxide dismutase (SOD), glutathione peroxidase (GSH-Px), and glutathione (GSH) are used to cope with free radicals in cell, SO_2_inhalation decrease activities of these enzymes and lead to lipid peroxidation may damage brain [41]. We did not find any association between PM_10_ and the risk of newly diagnostic ASD. It’s possible that PM_10_ was inversely correlated with CO level.

The study has several strengths. First, previous studies examined associations between air pollution and ASD by using cross-sectional or case-control study to estimate the effects of exposure to air pollution. The NHIRD covered 99% entire population in Taiwan which provide a sufficient, prospective, and population base database that allow us to test the relationship between air pollutants and ASD. In addition, we included 49,073 children hence the analysis approach may provide more statistical power than case-control design and provide benefits in studying rare outcome. Second, the longer follow-up period provide an opportunity to explore the risk of newly diagnostic ASD associated with postnatal exposure to long-term cumulative exposure. Third, this is the first study of the kind to be conducted in an Asian population. Race may be an important confounder that previous study had adjusted for this confounder in the relation between air pollution and ASD [4], [5]. This confounder may be neglected in Taiwan, because almost all residents in Taiwan are Han ethnicity. Our study also has several limitations. First, we were not be able to adjust for some confounders including occupational exposure, genetic factors, parental education, parental age, and birth weight [4], [5], [42]–[44], because there was no such information available in NHIRD. Second, we could not rule out possible misclassification of ASD. There errors are assumed to be non-differential with respect to lower exposure and higher exposure groups. Our nationwide population-based cohort study based on NHIRD has the advantage of having larger numbers of children which would reduce the uncertainly due to random error typical for smaller studies that collected detailed information on covariates. Third, previous studies have speculated that prenatal exposure to NO_2_ and O_3_ would disturb early neuromotor development, result in coordination deficits, reduce activity and reactivity, and affect neurobehavioral performances [45], [46]. Prenatal exposure to NO, NO_2_, PM_2.5_, PM_10_, and O_3_ were associated with an increased risk of autism [7], [47]. Duration of pregnancy can be seen as a sensitive period of ASD. Although, there was no detailed information on gestational age of children from NHIRD, we could not rule out the misclassification of prenatal exposure. Therefore, our primary objective is to assess the association between postnatal exposure to air pollution and the risk of newly diagnostic ASD.

In conclusion, the present study found a statistically significant association between O_3_, CO, and NO_2_ exposure and newly diagnostic ASD with an exposure-response pattern. Our finding suggests that improve ambient air quality, especially in traffic-related air pollutants and O_3_, might decrease the risk of newly diagnostic ASD.
