# Correction: Air Pollution and Newly Diagnostic Autism Spectrum Disorders: A Population-Based Cohort Study in Taiwan

**DOI:** 10.1371/journal.pone.0202996

**Published:** 2018-08-22

**Authors:** Chau-Ren Jung, Yu-Ting Lin, Bing-Fang Hwang

The risk increase in CO appears incorrectly twice in the article as per 10 ppb. The correct risk increase in CO should be per 100 ppb.

This error occurs in the penultimate sentence of the Abstract. The correct sentence is: The effect estimate indicating an approximately 59% risk increase per 10 ppb increase in O_3_ level (95% CI 1.42–1.79), 37% risk increase per 100 ppb in CO (95% CI 1.31–1.44), 340% risk increase per 10 ppb increase in NO_2_ level (95% CI 3.31–5.85), and 17% risk increase per 1 ppb in SO_2_ level (95% CI 1.09–1.27) was stable with different combinations of air pollutants in the multi-pollutant models.

The same error occurs in the second sentence of the first paragraph of the Discussion section. The correct sentence is: The effect estimate indicating an approximately 59% risk increase per 10-ppb increase in O_3_ level, 37% risk increase per 100-ppb in CO, 343% risk increase per 10-ppb increase in NO_2_ level, and 18% risk increase per 1-ppb in SO_2_ level was stable with different combinations of air pollutants in the two-pollutant models.
